# BamToCov: an efficient toolkit for sequence coverage calculations

**DOI:** 10.1093/bioinformatics/btac125

**Published:** 2022-02-23

**Authors:** Giovanni Birolo, Andrea Telatin

**Affiliations:** Medical Sciences Department, University of Turin, 10126 Turin, Italy; Gut Microbes and Health Programme, Quadram Institute Bioscience, Norwich Research Park, Norwich NR4 7UQ, UK

## Abstract

**Motivation:**

Many genomics applications require the computation of nucleotide coverage of a reference genome or the ability to determine how many reads map to a reference region.

**Results:**

BamToCov is a toolkit for rapid and flexible coverage computation that relies on the most memory efficient algorithm and is designed for integration in pipelines, given its ability to read alignment files from streams. The tools in the suite can process sorted BAM or CRAM files, allowing the user to extract coverage information via different filtering approaches and to save the output in different formats (BED, Wig or counts). The BamToCov algorithm can also handle strand-specific and/or physical coverage analyses.

**Availability and implementation:**

This program, accessory utilities and their documentation are freely available at https://github.com/telatin/BamToCov.

**Supplementary information:**

Supplementary data are available at *Bioinformatics* online.

## 1 Introduction

Sequencing coverage calculations have been done since the dawn of genomics (Lander and Waterman, 1988), commonly in relation to *a priori* theoretical calculations aimed at understanding the amount of effort required to produce sufficient DNA reads with capillary sequencers.

With the advent of massively parallel sequencing (also referred to as ‘next generation sequencing’) those *a priori* calculations began to be matched by *a posteriori* calculations made by mapping the DNA reads against a reference sequence (either a pre-existing reference, or the *de novo* assembly of the sequencing output itself). In this context some bases sequenced would not be accounted for (e.g. adaptor sequences, contaminants or unmappable reads).

When using paired libraries, it is also possible to evaluate the *physical coverage*, i.e. the number of times a base is spanned by a read pair.

There are already several tools to extract coverage information from alignment files (in BAM format): Samtools (Li *et al.*, 2009), Bedtools (Quinlan, 2014), Sambamba (Tarasov *et al.*, 2015) and the newer and more feature-rich Mosdepth (Pedersen and Quinlan, 2018b) and MegaDepth (Wilks *et al.*, 2021).

A common limitation of the existing tools is the inability to calculate physical coverage which is important when determining the integrity of assemblies using mate-pairs libraries. Also, it is not possible to separate the coverage per strand; if a position is covered only by forward reads, or only by reverse reads, it is probably due to misalignment. To address these limitations, we developed Covtobed (Birolo and Telatin, 2020), a simple yet efficient C++ program which, inspired by the UNIX philosophy of computer programming, focused on a single task supporting input and output streams.

Here we introduce the BamToCov suite of programs, implemented in the Nim language. BamToCov performs coverage calculations using an optimized implementation of the algorithm of Covtobed with new features to support interval targets, new output formats, coverage statistics and multiple BAM files, while retaining the ability to read input streams, thereby achieving an overall performance improvement (i.e., a smaller memory footprint and an increase in speed of up to 3×).

## 2 Materials and methods

BamToCov reimplements the coverage calculation algorithm of Covtobed, a C++ program, but with optimizations and using hts-lib (Bonfield *et al.*, 2021) for BAM parsing, via the hts-nim wrapper (Pedersen and Quinlan, 2018a) instead of libbamtools, hence natively supporting CRAM files.

The program and its companion utilities are written in Nim and tested using three compiler versions (1.2, 1.4 and 1.6).

Unlike other programs that allocate an integer array with the length of the reference sequence, BamToCov uses a streaming approach that takes full advantage of sorted input alignments. Furthermore, its memory usage depends only on the maximum coverage and not on the reference size.

The basic premise is that coverage is computed starting from zero at the leftmost base in each contig and updated on-the-fly while reading alignments and moving toward the right. Coverage is incremented at the start of each alignment and decremented at the end, keeping the ending positions in a priority queue. The suite of tools is automatically tested, and available via the BioConda project (The Bioconda Team *et al.*, 2018).

The scripts used to benchmark the execution times and the peak memory usage are available in the software repository.

## 3 Results

BamToCov, which can be used as a drop-in replacement for Covtobed, is faster and more memory efficient than Covtobed, while implementing a wide range of new features.

BamToCov is designed to support input streams and produce physical coverage and per-strand coverage calculations. BamToCov also enables the user to supply a set of intervals of interest (target) and use them both to restrict the output coverage to those regions and generate a table of statistics per interval. BamToCov supports targets in BED, GFF3 and GTF formats. Supplementary utilities provide support for computing read counts (rather than nucleotide coverage) and statistics over the whole chromosomes. An overview of the features is reported in Supplementary Note S1.

### 3.1 BamToCov performance

To evaluate the performance of BamToCov we adopted four test datasets: the genome sequencing of a yeast using short reads (SR) and long reads (LR), and two human datasets, a human exome, and a small targeted gene panel (see Supplementary Note S2 for a complete description and their availability).

BamToCov is reasonably fast, especially for datasets with few reads or long contigs (e.g. targeted gene panels); it is up to 2× faster than its predecessor (Covtobed), and very promising when evaluating the coverage of long reads (see Table 1 and Supplementary Note S3).

**Table 1.** Execution times and peak memory usage

Program	Fungus, SR		Fungus, LR		Exome		Gene Panel	
	*Mem*	*Time*	*Mem*	*Time*	*Mem*	*Time*	*Mem*	*Time*
BamToCov	2.7	9.77	4.3	85.4	5	24.09	2	0.35
Covtobed	4.0	16.45	5.0	162.7	6	33.81	4	0.53
Mosdepth	13.9	6.51	19.1	96.4	1983	3.02	6425	26.01
MegaDepth	11.6	5.97	11.6	107.9	995	10.14	980	9.24

*Note*: Peak memory usage (in megabytes) and average execution time out of 10 runs per program (seconds). Mosdepth was executed in fast mode.

BamToCov is the most memory efficient program for coverage calculations, with an improvement up to 50% when compared with the C++ implementation (Covtobed). Other programs relying on chromosome-sized vectors require up to 400× more memory to analyze a typical human exome, while BamToCov memory usage is not affected by the genome size (see Table 1 and Supplementary Note S4).

## 4 Conclusion

BamToCov is a program and suite of utilities engineered to simplify their application in bioinformatics pipelines requiring coverage calculations. It is designed to allow for flexible prototyping of bespoke pipelines, where the support for input and output streams and the low memory footprint can be valuable. For example, TraDIS-Xpress experiments (Turner *et al.*, 2020) rely on detecting uncovered regions across a large set of samples, and benefits from the availability of stranded reports.

In terms of performance, BamToCov proves to be a suitable alternative for gene panels and long reads datasets.

The peculiar algorithm adopted is the most memory efficient by far, and the new implementation in Nim yields further performance benefits both in terms of execution times and memory footprint.

## Funding

This work was supported by the Biotechnology and BiologicalSciences Research Counci (BBSRC; BB/R012490/1 and BBS/E/F/000PR10353, BB/R506552/1), the Medical Research Council (MRC; MR/T030062/1) and the European Union’s Horizon 2020 (GA101017598).

*Conflict of Interest*: none declared. 

## Supplementary Material

btac125_Supplementary_Data
