# Supplementary material for: BamToCov: an efficient toolkit for sequence coverage calculations
Source: Bioinformatics. 2022 Feb 23;38(9):2617–8. doi: 10.1093/bioinformatics/btac125 (PMC9048650; doi:10.1093/bioinformatics/btac125)
Supplement: btac125_Supplementary_Data [file btac125_supplementary_data.docx]

**Supplementary Notes**

**BamToCov, an efficient toolkit for sequence coverage calculations**

[**Note 1 - Features**](#_4o5l4pcj3c83) **2**

[BamToCov features](#_9cwfgxwakh7v) 2

[Nucleotide coverage analysis (BamToCov)](#_8ukwe4b7k3qp) 2

[Counting reads (BamToCounts)](#_ulj2uqdkigru) 3

[Target formats](#_9h3nsubapa1) 3

[Physical coverage](#_xik7cpthytls) 3

[**Note 2 - Datasets for the benchmark**](#_8ul2o675tbzm) **5**

[Specifications](#_50u0u1oef0gr) 5

[Availability](#_rgqevyfss70w) 5

[**Note 3 - Effect of read and genome length in execution times of BamToCov and other tools**](#_vb8xfedc6ynk) **6**

[Simulation of BAM files](#_q9uado1x5ia6) 6

[Effect of read length](#_nmfoifu3z0av) 6

[Effect of coverage](#_ye59iu4g31hh) 6

[Effect of target density](#_kdsd24krt7gh) 7

[Long reads targeted resequencing](#_d8eg2t3djb49) 8

[**Note 4 - Memory usage**](#_27azelfh90ao) **9**

[Methodology](#_yvt20qh40i9m) 9

[Effect of target density](#_4at8h11kc16o) 9

#

# Note 1 - Features

## BamToCov features

BamToCov is a redesigned and expanded suite based on Covtobed.

The core program features:

- Requires as input a sorted, but not indexed alignment file
- Support for input (BAM, CRAM) and output (BED, WIG) streams
- Physical coverage, and stranded coverage analysis
- Target support in BED, GFF3 and GTF formats

## Nucleotide coverage analysis (BamToCov)

Coverage analysis can be performed with diverse goals in mind and all the tools differs substantially in their implementation and ideal use case. MegaDepth for example has extended support for Wiggle traces and does not limit its input to alignments (BAM/CRAM files).

Here we compare the BamToCov 2.2.0 with three tools that can be used for base coverage calculations (BedTools 2.25.0, MegaDepth 1.1.2, Mosdepth 0.3.2).

|  | BamToCov | BedTools | MegaDepth | Mosdepth |
| --- | --- | --- | --- | --- |
| Requires sorted BAM | ✔ |  |  | ✔ |
| Requires indexed BAM |  |  |  | ✔ |
| Input stream | ✔ |  |  |  |
| Output stream | ✔ | ✔ | ✔ |  |
| Coverage BED Graph | ✔ | ✔ | ✔ | ✔ |
| Physical Coverage | ✔ |  |  |  |
| Stranded Coverage | ✔ |  |  |  |
| CIGAR Operations |  |  | ✔ | ✔ |
| Fixed step WIG output | ✔ |  |  |  |
| Variable step BigWig output |  |  | ✔ |  |
| Variable step WIG output |  |  | ✔ |  |
| BED Target | ✔ | ✔ | ✔ | ✔ |
| GFF3 Target | ✔ |  |  |  |
| GTF Target | ✔ |  |  |  |

## Counting reads (BamToCounts)

BamToCounts can be used when a digital count of sequences per interval is required, instead of the per nucleotide coverage. The possibility to count separately forward and reverse counts enables its use in pipelines for the analysis, for example, of TraDIS-Xpress experiments, where digital counts of insertion sites are evaluated in a strand-specific manner.

BamToCounts reads unindexed BAM files and supports BED, GFF3 and GTF annotations, sharing the same parsing and feature intersection methods of BamToCov. The current implementation does not require sorted BAM files as input.

## Target formats

The target (set of features of interest in the reference genome) is internally represented as a table of chromosome names and a sequence of intervals, defined having a start and stop position (0-based, start inclusive and stop exclusive) and an interval name. If the interval name is not present, it is produced concatenating chromosome name and start/stop coordinates (*e. g.* chr1:1020-1500).

Tests are performed to ensure a coherent representation of the targets from the three supported input formats: BED (<https://genome.ucsc.edu/FAQ/FAQformat.html#format1>), GFF3 (<http://gmod.org/wiki/GFF3>) and GTF (<http://gmod.org/wiki/GFF2>). Recently (see <https://github.com/samtools/hts-specs/pull/570>) a BED format formal specification has been proposed.

## Physical coverage

When using paired libraries, we can also evaluate the physical coverage, the average number of times a base is spanned by a read pair. Figure 1 depicts the difference between sequence and physical coverage (Figure S5.1).

The analysis of physical coverage allows understanding if the library, rather than the sequencing reads, sampled a region. If the physical coverage drops unexpectedly, this can be an indication of a misassembled region, hence the possibility to quickly identify variations in its value can be beneficial.

BamToCov in physical coverage mode (--physical) will increase the coverage from the beginning of the leftmost alignment to the end of the rightmost, *i.e.* including both the reads and the inter-reads space (Figure S5.2).

##
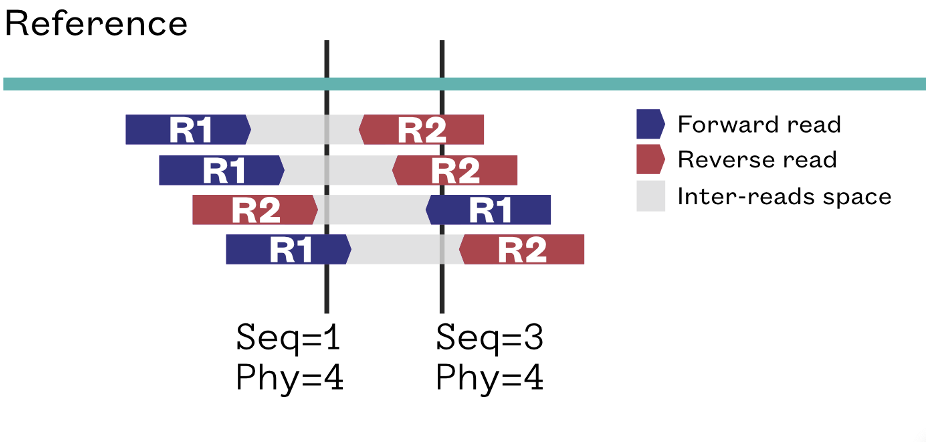


**Figure S5.1** – Schematic representation of paired-end reads (blue and purple arrows) mapped against a reference sequence (red). The sequence coverage (Seq) and the physical coverage (Phy) of two nucleotide coordinates (black lines) have been calculated, showing the difference between the two.


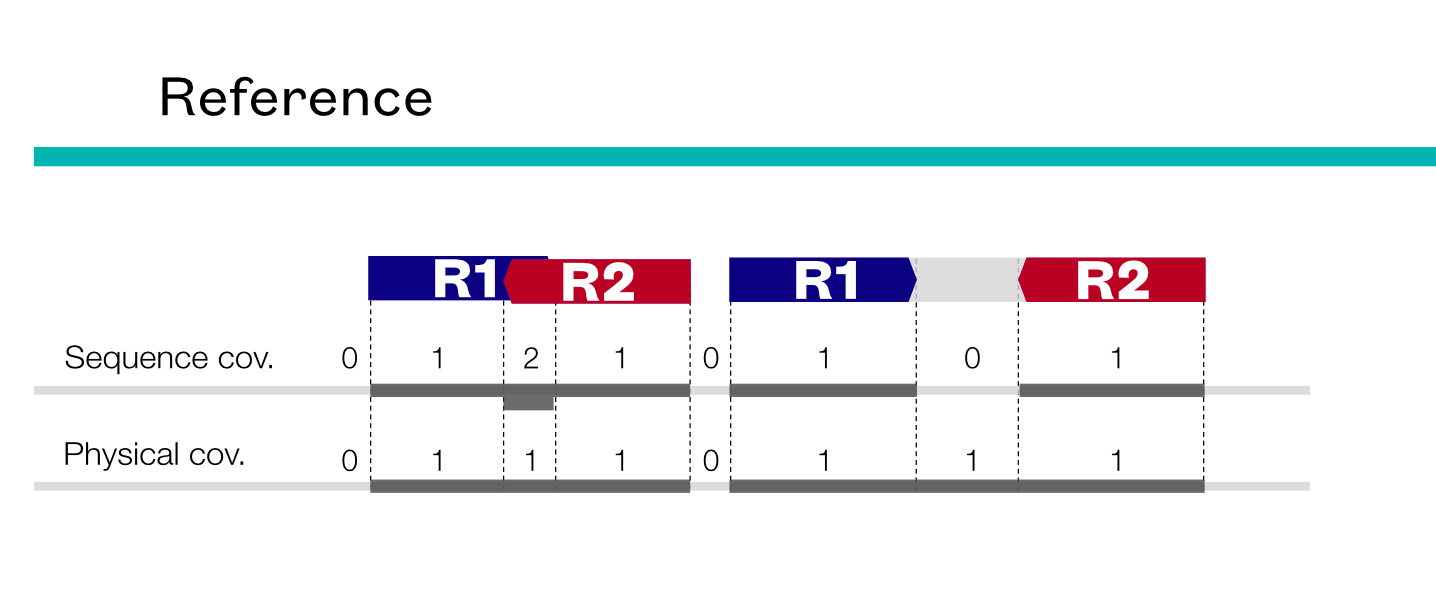


**Figure S5.2** – Schematic representation of the calculation of physical and sequence coverage in the case of overlapping (*left*) and non-overlapping (*right*) paired reads.

# Note 2 - Datasets for the benchmark

## Specifications

To evaluate the speed and memory usage in different scenarios, we adopted four datasets:

- **Fungus SR (Short Reads)**: whole-genome shotgun of Short Reads produced via Illumina MiSeq sequencing, mapped against the assembly of an isolate of Candida albicans
- **Fungus LR (Long Reads)**: whole-genome shotgun of Long Reads, produced via Nanopore sequencing, mapped against the same genome as SR
- **Human Exome**: Illumina sequencing of sample HG00258 from the 1000 Genomes project and
- **Human Gene Panel**: Illumina sequencing of a Human target enrichment panel of 16 genes.

**Table S2** - Specifications of the BAM files in terms of reference (Number of sequences, maximum length and N50) and reads mapped (Number of mapped reads, maximum length and N50).

| **Name** | **Reference** | | | **Reads** | | |
| --- | --- | --- | --- | --- | --- | --- |
|  | **#Seqs** | **Max. Len.** | **N50** | **#Mapped** | **Max. Len.** | **N50** |
| **Fungus SR** | 60 | 1,161,538 | 720,776 | 9,174,524 | 151 | 151 |
| **Fungus LR** | 60 | 1,161,538 | 720,776 | 35,005 | 64,087 | 10,411 |
| **Human Exome** | 3,366 | 248,956,422 | 145,138,636 | 211,412,837 | 76 | 76 |
| **Human Gene Panel** | 25 | 249,250,621 | 155,270,560 | 437,393 | 151 | 150 |

## Availability

The datasets (BAM files) have been deposited to Zenodo:

Telatin, Andrea. (2021). Alignment files for coverage benchmarks: Illumina and Nanopore sequencing datasets (1.0) [Data set]. Zenodo. <https://doi.org/10.5281/zenodo.5636944>

#

# Note 3 - Effect of read and genome length in execution times of BamToCov and other tools

## Simulation of BAM files

A program to generate random BAM files having a single reference sequence of variable length *genL*, and read length randomly generated at each position of the reference and with length *minL* to *maxL* to produce a total coverage *C* is provided with the repository under **scripts/simulate-long-bam.py**. The program can either produce perfectly aligned reads, or simulate a random CIGAR with INDELs.

## Effect of read length

Two datasets have been generated against a 100 Mbp genome, one with long reads (1,000 to 10,000 bp) and one with short reads (100 to 300 bp).

We refer to short reads and long reads as SR and LR, respectively. Considering that the coverage is constant, the *number of alignments* is higher in the short reads dataset, from 90,621 to 2,506,316 (~30X increase).

Execution times, in seconds, as measured with *hyperfine*.

**Table S2.1** Execution times (seconds) measured using a simulated 100 Mbp genome and, respectively, long reads (LR) and short reads (SR).

|  | LR 100 Mbp | | SR 100 Mbp | |  |
| --- | --- | --- | --- | --- | --- |
| **Command** | **Mean** | **St. dev.** | **Mean** | **St. dev.** | **Increase** |
| megadepth | 1.954 | 0.169 | 5.552 | 0.334 | 2.8 |
| bamtocov | 1.617 | 0.106 | 21.147 | 0.362 | 13.1 |
| covtobed | 4.703 | 0.066 | 25.036 | 0.788 | 5.3 |
| mosdepth | 3.873 | 0.437 | 10.778 | 0.894 | 2.8 |

## Effect of coverage

Here we compare a 10Mbp genome in 50X vs 200X shotgun (long reads). All the tools show a linear increase in calculation times given a 4X increase in coverage.

**Table S2.2** Effect of genome coverage in the execution times of nucleotide coverage calculation using a simulated alignment file with 50X and 200X average coverage, respectively.

|  | 50X | | 200X | |  |
| --- | --- | --- | --- | --- | --- |
| **Command** | **mean** | **stddev** | **mean** | **stddev** | **Increase** |
| megadepth | 0.71 | 0.06 | 2.38 | 0.03 | 3.3 |
| bamtocov | 0.76 | 0.06 | 3.11 | 0.28 | 4.1 |
| covtobed | 2.30 | 0.03 | 9.36 | 0.13 | 4.1 |
| mosdepth | 0.96 | 0.03 | 2.88 | 0.06 | 3.0 |

## Effect of target density

To assess the effect of target density we simulated a set of BAM files having a single chromosome (100 Mbp) and a total target size of 20 Mbp with a total of 1M alignments, but distributed among a single feature of 20 Mbp, or multiple features (2, 4, 8 and 16 respectively).

The simulated BAM files were produced with a script present in the repository:

./scripts/benchmarking/make-bam-targeted.py \

-l 100M -n 1M -t 20M -f $FEAT -m 100 -M 300 -o _test/targets/20M_${FEAT}feat.bam

Target density did not affect the performance of MegaDepth, the fastest tool in this comparison. BamToCov (2.3X slower) showed a negative correlation between the number of features and speed, while MosDepth in fast mode (2.5X slower) showed a positive correlation, which can be explained by the use of indexed files (index only required by Mosdepth).

**Table S2.3** Execution times (seconds) to evaluate the effect of uneven reads distribution across a genome using simulated targets with increasing number of features.

| **Command** | **Features** | **mean** | **stddev** |
| --- | --- | --- | --- |
| bamtocov $file | 1 | 3.5 | 0.1 |
| megadepth $file --coverage |  | 1.6 | 0.1 |
| mosdepth -x pref $file |  | 4.1 | 0.2 |
| bamtocov $file | 2 | 3.6 | 0.1 |
| megadepth $file --coverage |  | 1.6 | 0.0 |
| mosdepth -x pref $file |  | 4.1 | 0.1 |
| bamtocov $file | 4 | 3.7 | 0.2 |
| megadepth $file --coverage |  | 1.6 | 0.0 |
| mosdepth -x pref $file |  | 4.2 | 0.1 |
| bamtocov $file | 8 | 4.0 | 0.5 |
| megadepth $file --coverage |  | 1.6 | 0.1 |
| mosdepth -x pref $file |  | 4.0 | 0.0 |
| bamtocov $file | 16 | 3.7 | 0.1 |
| megadepth $file --coverage |  | 1.6 | 0.0 |
| mosdepth -x pref $file |  | 3.8 | 0.1 |

## Long reads targeted resequencing

BamToCov performs well on targeted resequencing experiments, as shown in the “Human Gene Panel” dataset, and on long reads (“Fungus, LR”). We download a single sample from the Short Reads Archive (SRR13615770) that was performed via targeted sequencing using the Oxford Nanopore platform. It was aligned against the human genome and the output BAM file analysed with BamToCov, MegaDepth and Mosdepth, with 5 runs per test.

**Table S2.4** Execution times (seconds) of different tools analysing sample SRR13615770.

| **Command** | **Mean** | **Min** | **Max** | **Relative** |
| --- | --- | --- | --- | --- |
| bamtocov $FILE | 32.357 ± 0.261 | 32.172 | 32.541 | 1.00 |
| megadepth --coverage --longreads $FILE | 46.115 ± 0.954 | 45.440 | 46.789 | 1.43 ± 0.03 |
| mosdepth -x /tmp/file $FILE | 92.359 ± 5.448 | 88.506 | 96.211 | 2.85 ± 0.17 |

#

# Note 4 - Memory usage

## Methodology

Peak memory usage has been measured with a Bash script as reported in the documentation of the programme: <https://telatin.github.io/bamtocov/notes/memory.html>. See supplementary note 2 for the datasets used.

**Table S3.1** Peak memory usage of four coverage tools against four typical datasets (Kilobytes)

| **Program** | **Fungus, SR** | **Fungus, LR** | **Human Exome** | **Human Gene Panel** |
| --- | --- | --- | --- | --- |
| BamToCov | 2,740 | 4,376 | 5,700 | 2,172 |
| Covtobed | 4,080 | 5,008 | 6,588 | 4,052 |
| Mosdepth | 13,952 | 19,140 | 1,983,928 | 6,425,744 |
| MegaDepth | 11,644 | 11,636 | 995,232 | 980,040 |

## Effect of target density

To assess the effect of target density we simulated a set of BAM files having a single chromosome (100 Mbp) and a total target size of 20 Mbp with a total of 1M alignments but distributed among a single feature of 20 Mbp, or multiple features (2, 4, 8 and 16 respectively).

​​Results (below) show a constant memory usage, where MegaDepth requires 100X memory if compared with BamToCov and Mosdepth requires 200X more memory.

**Table S3.2** Peak memory usage of using simulated alignment files with a constant number of reads (1M) and uneven distribution in a simulated target with increased number of features (total target size 20 Mbp, total genome size 100 Mbp)

|  | BamToCov | MegaDepth | Mosdepth |
| --- | --- | --- | --- |
| 20M_1feat.bam (1 feature) | 3,608 | 397,284 | 785,596 |
| 20M_2feat.bam (2 features) | 3,608 | 397,356 | 785,532 |
| 20M_4feat.bam (4 features) | 3,792 | 397,396 | 785,620 |
| 20M_8feat.bam (8 features) | 3,772 | 397,280 | 785,724 |
| 20M_16feat.bam (16 features) | 3,748 | 396,872 | 785,776 |

# 
